# Michigan

**Published:** 1897-08

**Authors:** 


*Michigan.* The State Legislature of this State failed to pass the bill intended to establish a board of veterinary medical examiners and better regulate the practice of veterinary science in this State. The appointment by the Governor of Dr. Geo. Coester, of Detroit, Mich., as State Veterinarian does not seem to have met with a very warm approval by the veterinary profession throughout the State or those connected with the State Association.

